# Supplementary figures and images for: Helicobacter pylori diversification during chronic infection within a single host generates sub-populations with distinct phenotypes
Source: PLoS Pathog. 2020 Dec 28;16(12):e1008686. doi: 10.1371/journal.ppat.1008686 (PMC7794030; doi:10.1371/journal.ppat.1008686)

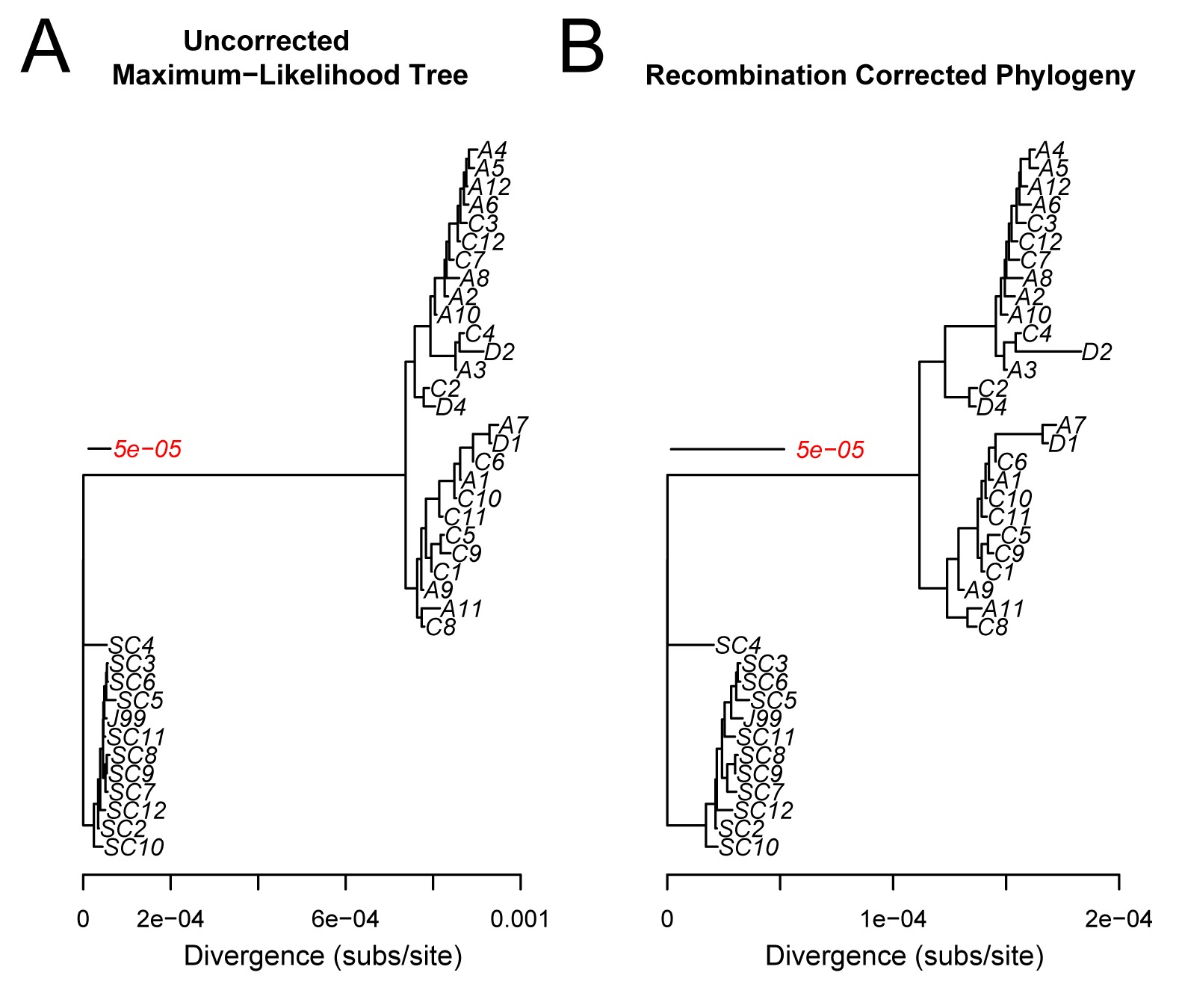

Supplement: S1 Fig — X-axis represents divergence from common ancestor (substitutions/site) and scale bar with length indicated in red text is shown. Scale of branches is indicated in red. (A) The maximum likelihood tree constructed in Nextstrain (Fig 4A, <https://nextstrain.org/community/salama-lab/Hp-J99>) and (B) the recombination corrected phylogeny created in ClonalFrameML with rescaled branch lengths are shown. Tree construction is described in detail in the methods. (TIF) [file ppat.1008686.s001.tif]

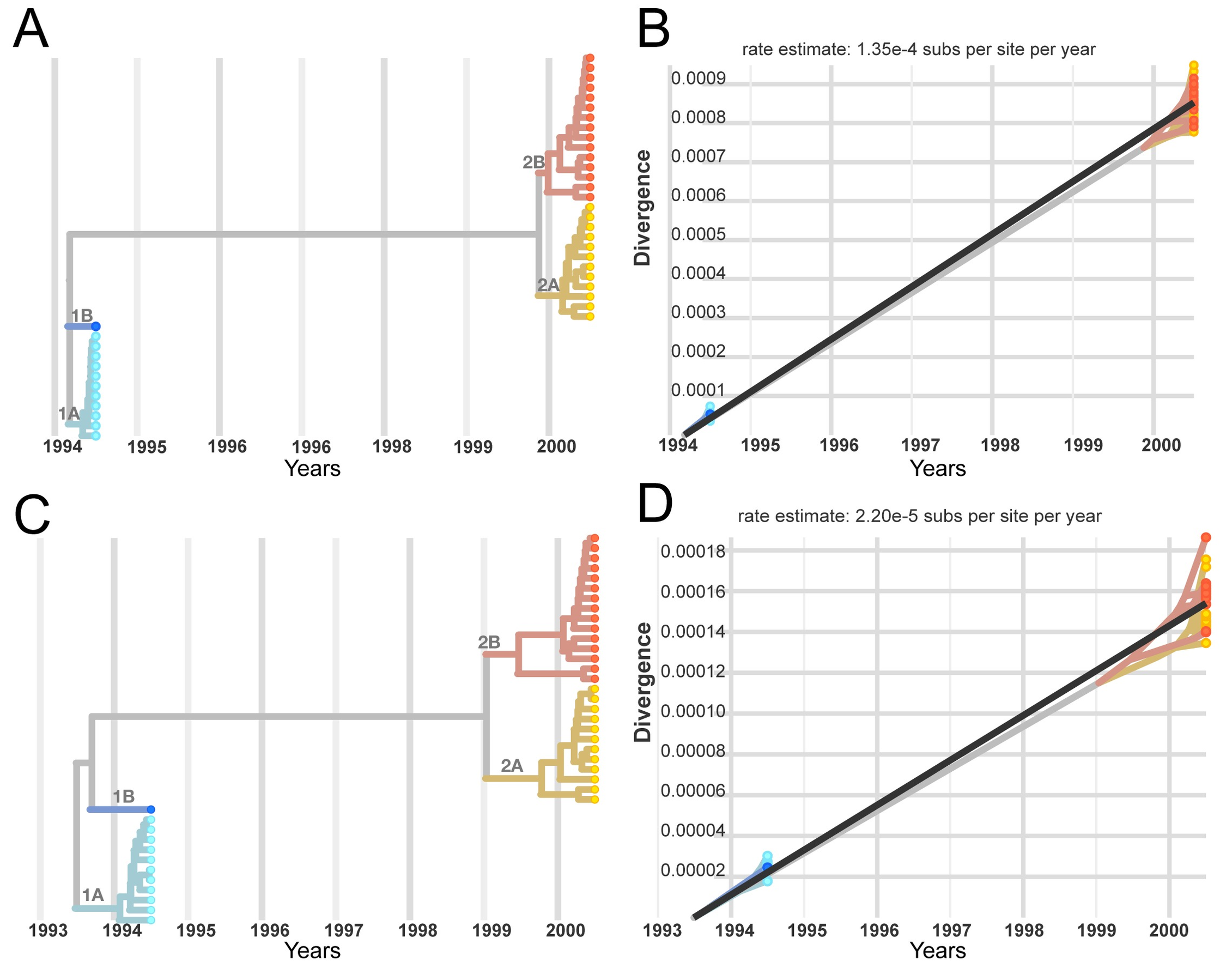

Supplement: S2 Fig — X-axis indicates the date in years and Y-axis (B, D) indicates divergence (substitutions/site) from the ancestral root. (A) A time-scaled version of the maximum likelihood tree with (B) molecular clock rates inferred from the root-to-tip distance using TreeTime [75]. (C-D) Shows time-scaled tree (C) and inferred molecular clock for the recombination corrected tree (D). (TIF) [file ppat.1008686.s002.tif]

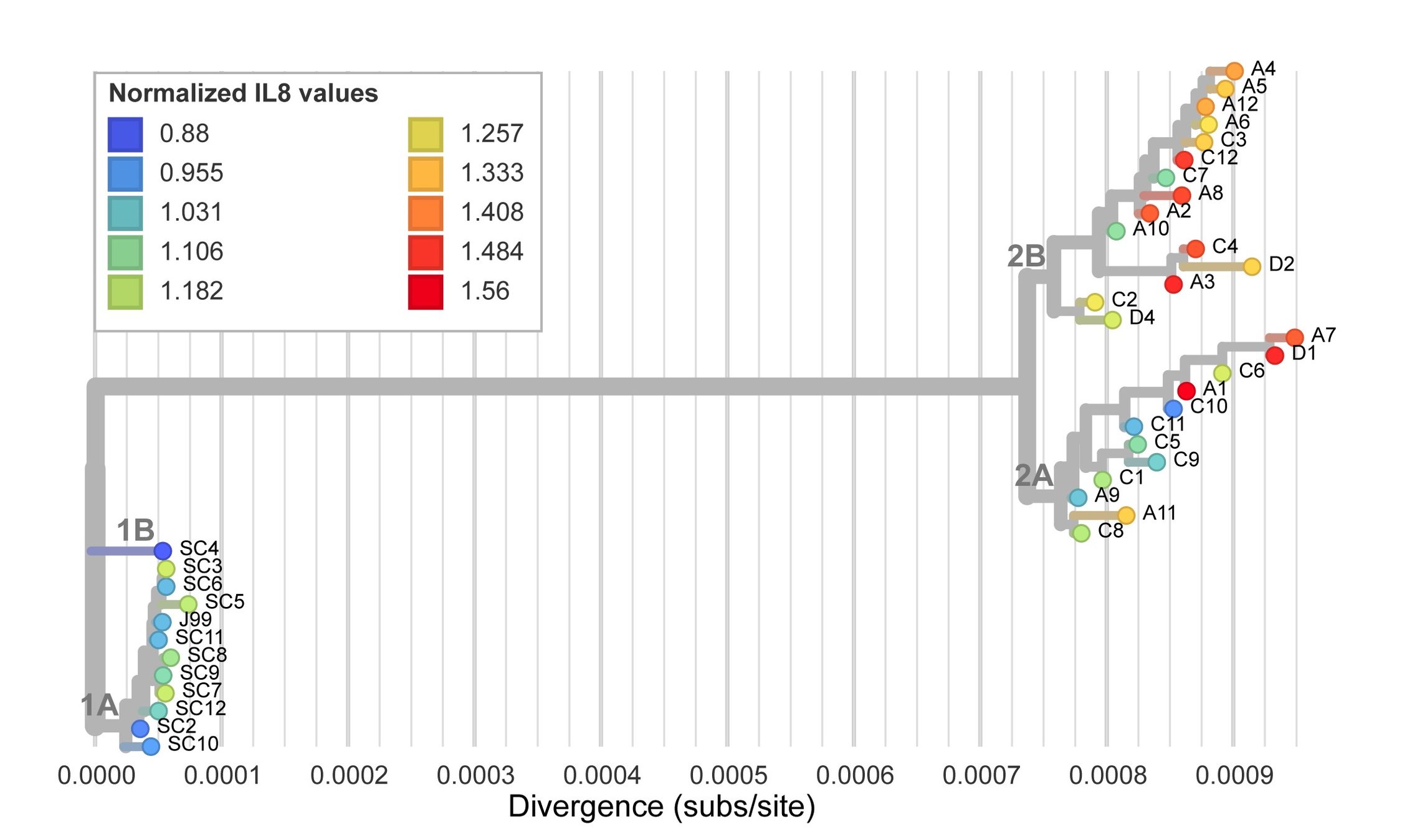

Supplement: S3 Fig — Maximum likelihood tree overlaid with inflammatory cytokine, IL-8, secretion phenotype after 24 hours of co-culture (MOI = 10) with gastric epithelial cell line (AGS). Leaf colors represent normalized IL-8 induction relative to ancestral isolate J99 for each isolate as shown in the figure legend. (TIF) [file ppat.1008686.s003.tif]

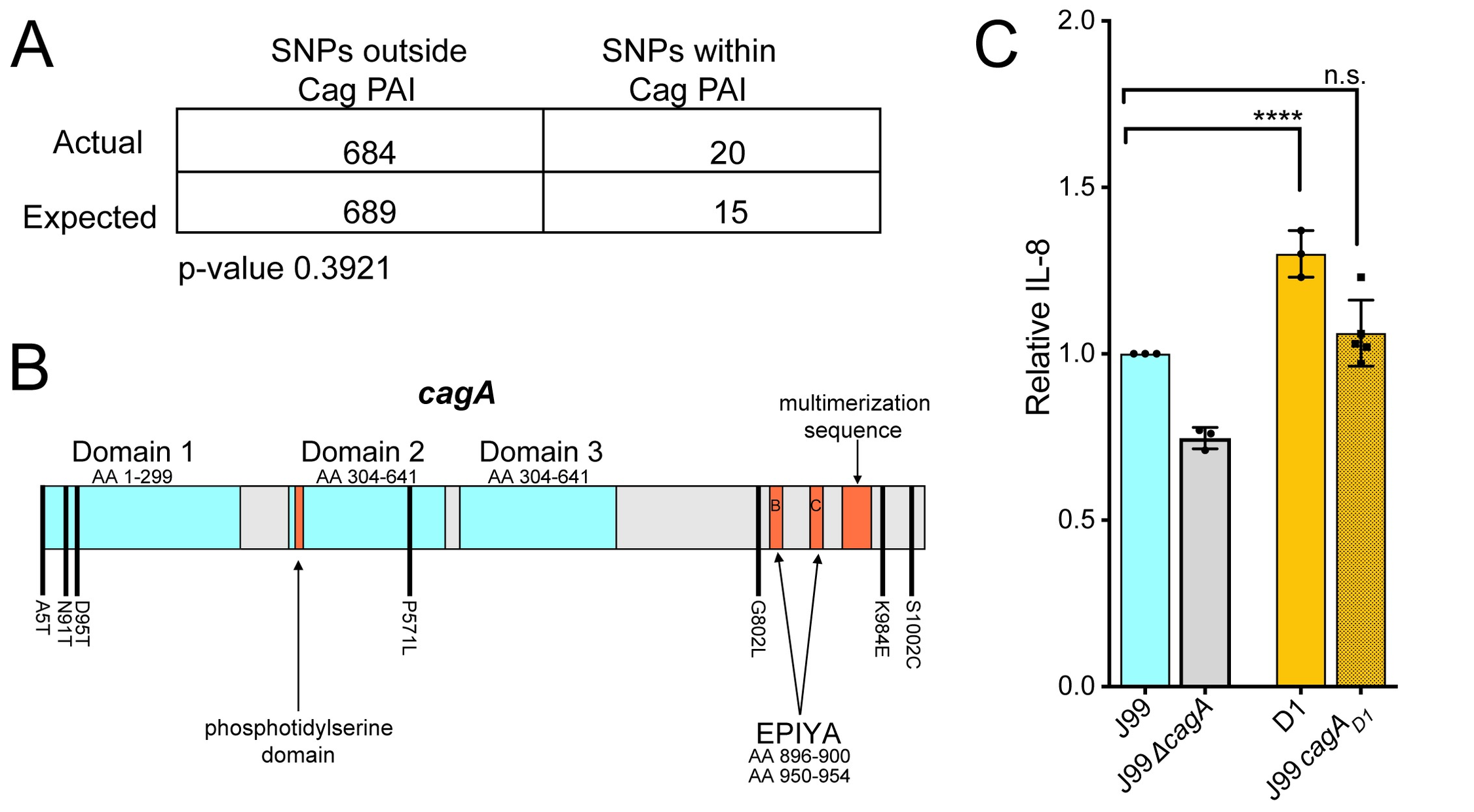

Supplement: S4 Fig — (A) Contingency table of nSNPs falling within and outside Cag PAI compared to expected values based on a normal distribution. Significance was determined using a Fisher’s exact test.(B) CagA gene schematic labeled with nonsynonymous amino acid changes shared by all recent isolates (black bars). The three protein domains identified in the published crystal structure (blue), including the flexible N-terminal region (Domain I, amino acids 1–299), the anti-parallel beta sheet (Domain II, amino acids 304–641), and the N-terminal binding sequence (Domain III, amino acids 304–641) are labeled. Known host protein interaction motifs including the integrin binding phosphotidylserine domain, phosphotyrosine EPIYA sites, and multimerization sequence are also labeled in orange [79]. (C) Levels of IL-8 produced by cagA allelic exchange strains relative to J99 24 hrs post infection of AGS cells (MOI = 10). Data points represent averaged values from triplicate wells from at least 3 independent biological replicates. Significance was determined with a one-way ANOVA with Dunnett’s corrections (n.s., not significant; **** p<0.0001). (TIF) [file ppat.1008686.s004.tif]

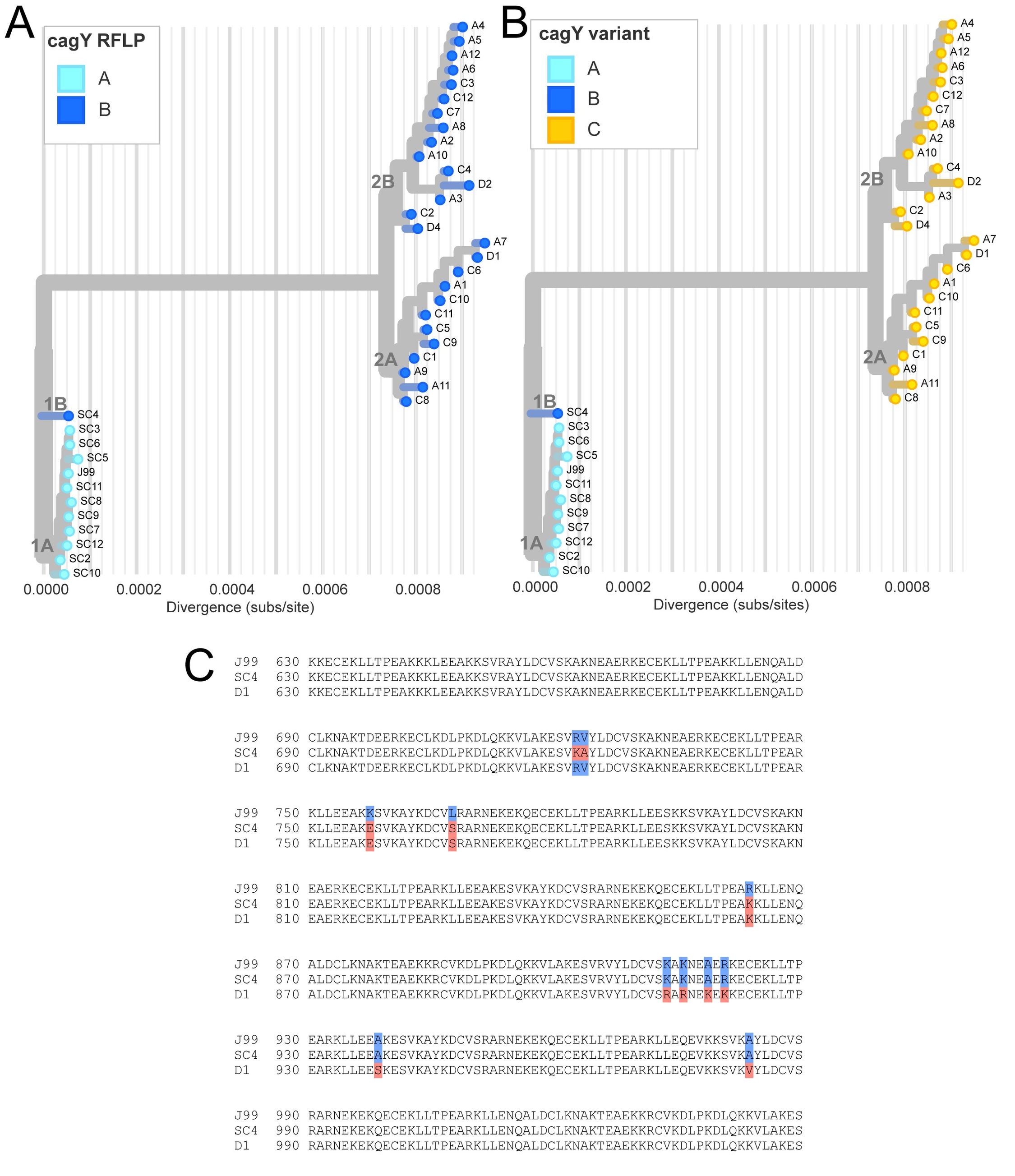

Supplement: S5 Fig — (A) Maximum likelihood tree overlaid with two different cagY RFLP subtypes detected with restriction enzyme DdeI. RFLP subtypes, named A and B according to the figure legend, are shown in Fig 6A. (B) Maximum likelihood tree overlaid with unique cagY alleles detected with Sanger sequencing. Leaf colors correspond to each of the three unique alleles detected and reported in Fig 6B. Group 1A shares allele A, group 1B shares allele B, and groups 2A and 2B share allele C. (C) Amino acid alignment of multiple repeat regions of three representative cagY alleles detected in the collection. J99 represents the allele found in subgroup 1A (allele A), SC4 represents the allele found in subgroup 1B (allele B), and D1 represents the allele found in 2A and 2B (allele C). Polymorphic sites are highlighted with amino acids in blue representing the reference (J99, AE001439) and red indicating a nonsynonymous substitution. (TIF) [file ppat.1008686.s005.tif]

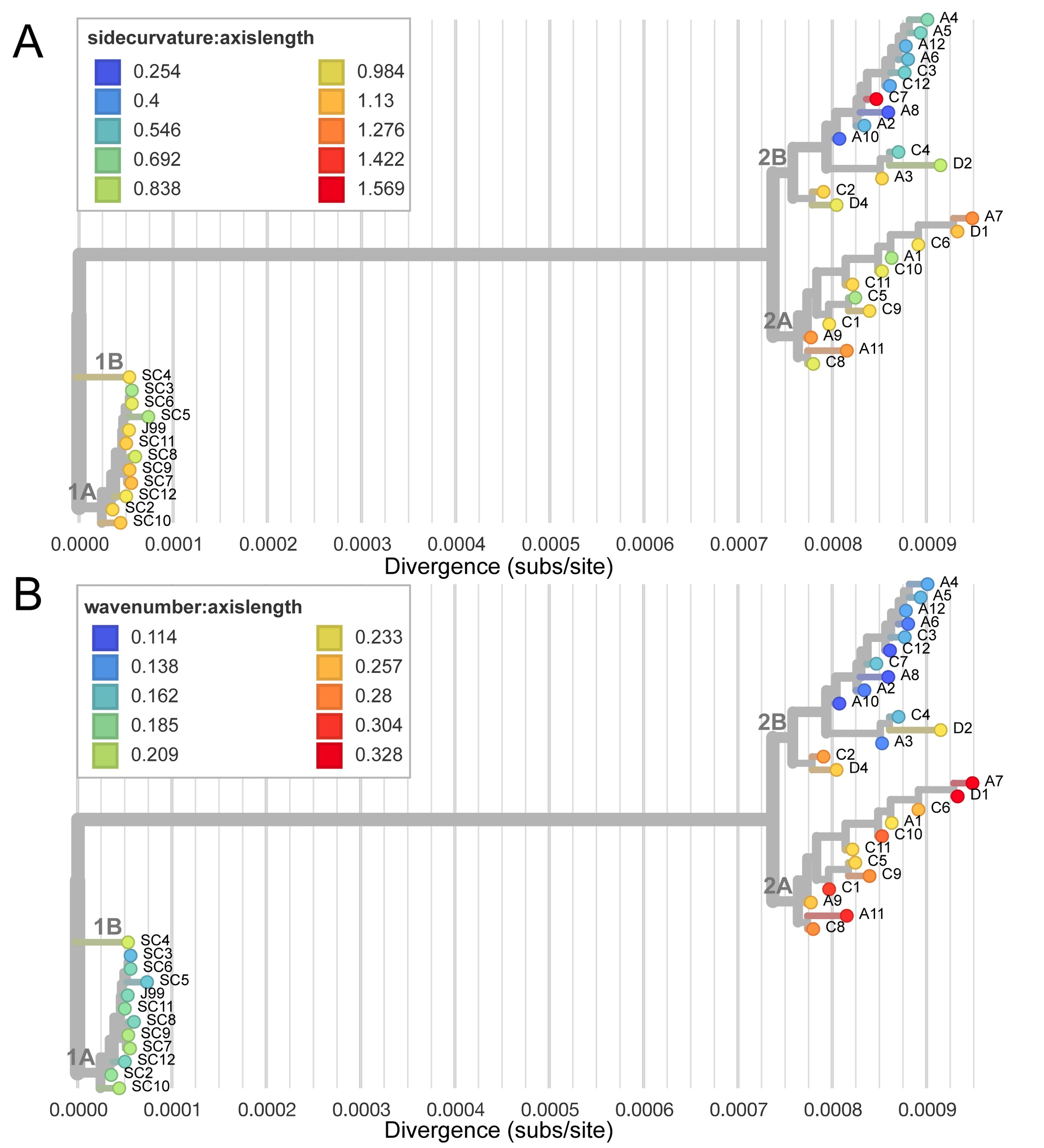

Supplement: S6 Fig — Maximum likelihood tree overlaid with cell shape measurements taken from 2-D phase contrast images using CellTool. Leaf colors represent side curvature normalized by centerline axis length (A) or wave number normalized by centerline axis length (B) as indicated in the figure legends. (TIF) [file ppat.1008686.s006.tif]

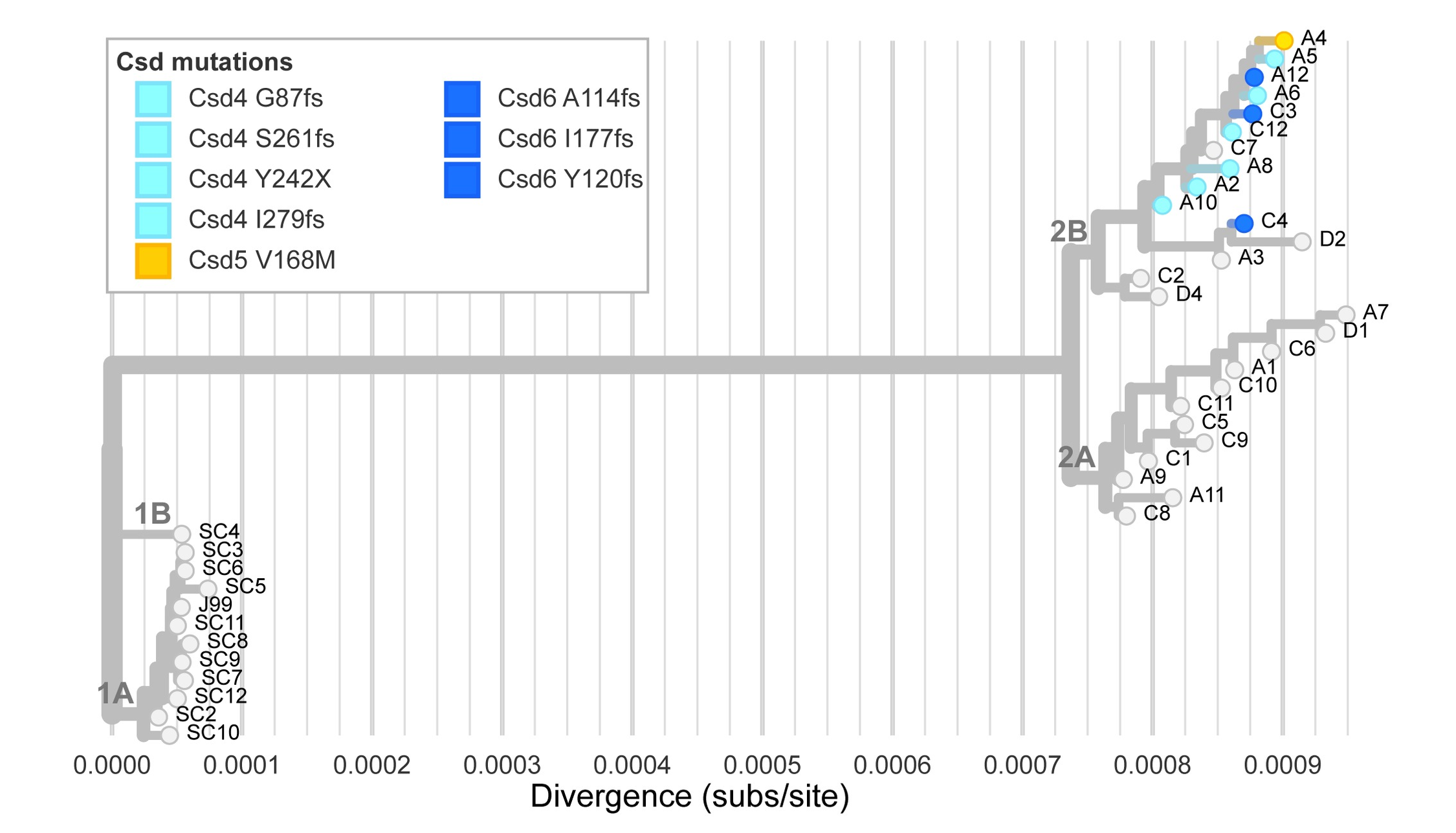

Supplement: S7 Fig — Maximum likelihood tree labeled with putative loss of function mutations in cell shape determining genes (csd). Leaf colors indicate mutations in csd4 (light blue), csd5 (yellow), csd6 (dark blue) listed in the figure legend with amino acid mutations. All isolates that have retained helical shape are in gray. (TIF) [file ppat.1008686.s007.tif]
