# Supplementary material for: Helicobacter pylori diversification during chronic infection within a single host generates sub-populations with distinct phenotypes
Source: PLoS Pathog. 2020 Dec 28;16(12):e1008686. doi: 10.1371/journal.ppat.1008686 (PMC7794030; doi:10.1371/journal.ppat.1008686)
Supplement: Attachment — Submitted filename: 2020_09_15_Response_Letter.docx [file ppat.1008686.s017.docx]

We would like to thank the reviewers for their insightful comments on our manuscript. The major concerns related to a desire for additional description of the genetic diversity measured in this study, comparisons with other studies and phylogenetic implications. Below is a description of changes made to the manuscript. Additional data for the reviewer’s consideration are included in line with the text. Revised figures and tables as well as new supplemental figures that we have added to the manuscript are included at the end of the document.

Major Revisions suggested by Reviewers 2 and 3

Reviewer #2:

1) **Overall the introduction describes an array of mechanisms of H pylori variation but not a sense of what is known about the typical diversity within or among infections. Please provide this background and relate your results to what’s been shown.**

**Response:** We have expanded the introduction to include additional background on the extent of within-host diversity at lines 121-131:

“*Whole genome sequencing of sequential isolates from individual infections detected accumulation of point mutations and frequent recombination among co-colonizing isolates. Within host diversity is acquired in a clock-like manner with average estimated mutation rate of 2.5x10^-5^ substitutions per site per year. However, in the majority of individuals recombination is the main driver of within-host diversification, which introduces changes at a median rate of 5.5 × 10^−5^ substitutions per initiation site per year [45,46]. The rate of polymorphisms introduced via recombination compared to mutation (r/m rates) vary widely between individuals and likely depends on the presence of multiple co-infecting strains [47,48]. Phylogenetic studies of isolates within a single individual, have observed signatures of diversifying selection in support of selective pressures during chronic stomach colonization [7,37,46]*.”

2) **“Methods and rigor of sequencing (what was average coverage, estimated genome size per isolate?) and variant calling require clarification.”**

**Response:** The coverage metrics for each genome is reported on the Github page (<https://github.com/salama-lab/Hp-J99/blob/master/data/seq-metadata-2020.xlsx>). This table has been moved to the supplement (Table S8) so it can more easily be accessed. Approximate genome size was never calculated because we did not build de novo assemblies in this analysis; we only mapped to the reference (J99, AE001439). However, we did calculate the number of shared sites (n) between pairwise comparisons, which eliminated missing or ambiguous sites. These values are reported in Table S5. Changes have been made to the Materials and Methods at lines 542-546 and the section at line 571-576 to clarify:

*“J99 ancestral as the reference strain (AE001439), variants were called from raw paired end reads using the Breseq v0.35.0 software with default parameters and SNPs were further validated using default Samtools software suite [69]. Four isolates with average coverage below 30x were dropped from the analysis.* *Of the remaining 39 isolates, 34 had coverage >100x. Additional sequencing metrics can be found in Table S8.”*

*“The number of nucleotide differences per site (genetic distance) between pairs of isolates was calculated using PopGenome (R, v 4.0.2) using the merged sample variant call file (VCF) file available on Github (nucleotide diversity,* π*, [71], PopGenome, [72]). All sites that did not align to the reference genome, J99, or had read depth <5 were excluded from the analysis, which removed missing or ambiguous sites resulting from structural variation or mis-mapping.”*

3) **While breseq is a standard-bearer, the key question is how many sites are informative – since some are prone to variation regardless of variant calling method even for extremely well studied reference genomes -- and how many of those remaining can be used to rigorously differentiate isolates.**

**Response**: In this analysis we chose to include variation across all genes for our pairwise comparisons and creation of the tree/dendrogram. We acknowledge not all of these sites are informative for discerning evolutionary relationships between isolates. However, for our purposes, these sites allowed us to identify genetic variants underlying the distinct bacterial phenotypes. We did refine Table 1 to reduce the number of SNPs reported as requested by reviewer 3. Please also see the new Table 1 and our comments to reviewer 3, bullet #2.

_____________________________________________________________________

**Response to reviewer 3, bullet #2**: We found 625 SNPs and 130 Indels in our J99 isolate that differ compared to the published reference of J99 (AE001439). A total of 553 of these polymorphic sites were common to all isolates in the collection. We agree with reviewer 3 that including these in Table S1A/Table 1/Text L189 gives an inflated number of ‘total unique SNPs’ and can lead to misinterpretation of the diversity present. This was also expressed by reviewer 2. We have modified Table 1 and Table S1a-b so that variable sites between our J99 isolate and reference that are shared across all isolates were not included in the overall total count. Common SNPs and Indels are now listed in two separate supplementary tables Table S1c and Table S1d. Values have also been changed in the text (L37, L181). Figure 3B and Table S3 have also been altered to reflect the edited SNP total. We have acknowledged the variation in our J99 isolate sequence compared with the published GenBank reference at line 171-174:

“*Our J99 isolate differed from the reference J99 (AE001439) at 755 polymorphic sites (625 SNPs, 130 indels); of these 553 polymorphisms were common to all isolates in the collection (Table S1c-d) and were excluded from downstream analysis*.”

For the “recent SNPs” part of Table 1, only SNPs absent from ancestral population were counted and reported. Therefore, the diversity reported is not inflated (Total=1,379 SNPs).

___________________________________________________________________

4) ***conclusions are drawn from two sampling periods with no intermediates, and the manuscript relies upon, and often overstates, correlations drawn from only these two periods. This must be acknowledged and reconciled*.**

**Response:** While we agree that additional time points and samples would give even more information, we have obtained valuable information regarding phenotypic and genetic variation with the samples that we have. While there are no intervening samples available for analysis from this subject, we have added additional analysis that allow us to better draw comparisons to other published studies that analyzed isolates from the same individual at different time points (lines 434-442).

“*Others have estimated the within-host mutation rate to be between 6.5-0.5 x 10^-5^ substitutions per site per year by excluding predicted recombination sites in order to make evolutionary inferences [35,44,47,54]. However, it remains debated whether diversity generated via recombination versus mutation can be accurately identified and filtered to reconstruct evolutionary relationships [56,57,58]. Since clustered nucleotide polymorphisms (CNPs), typically excluded from molecular clock rate calculations, were included in this analysis, our overall within-host molecular clock rate of 1.3 x 10^-4^ subs/site/yr is elevated compared with rates based on substitutions from mutation alone [44].”*

We have also reanalyzed our data using tools used in these cited studies such that our observations can be more easily compared to other studies, as was requested by reviewer 3 (lines 268-282; Fig S1 and S2).

“*The inferred molecular clock rate calculated from a time-scaled tree using the root-to-tip distance (1.35 x 10 ^-4^ subs/site/year) is similar to the molecular clock rate calculated from pairwise comparisons (Fig. 2a, Fig. S2a-b). Expectedly, this rate is much higher than other within-host estimates that exclude predicted recombination events [45]. To explore the impact of recombination on the tree branch scale and topology, we generated a recombination corrected phylogeny using ClonalFrameML (Fig. S1b). We found the branch lengths of the maximum likelihood tree (Fig. 4a, Fig. S1a) were on average 5 times greater than that of the recombination corrected tree, but general topology remained the same (Fig. S1b). The inferred molecular clock rate from the recombination corrected time-scaled tree was 2.2 x 10^-5^ subs/site/year, which is consistent with published estimates (Fig. S2d) [45,48,54]. We estimated a time to most recent common ancestor (TMRCA) of 1 year for the ancestral population and 1.5 years for the recent population. Low TMRCA are typically observed in phylogenetic studies of within host evolution of H pylori; and in the absence of antibiotic treatment, this is thought to occur as a result of immune pressure [37,48]*”

While our study indeed has limitations that are directly acknowledged in the manuscript (lines 411-412, 504-506, 510-512), we hope these comparisons will allow readers to assess our results in the context of other studies on this topic.

“*Together these results suggest that there are additional, unknown properties of these isolates contributing to colonization (Fig. 9c).*”

“*Robust colonizers may be more likely to be involved in person-to-person transmission in humans, but it is also possible that these strains may behave differently in other animal models or human hosts*.”

“*Further exploration of the genetic basis for mouse colonization advantage using the subgroup specific variants defined in this study may give new clues to the complex selective forces operant during chronic stomach colonization by H. pylori.”*

5) **Sampling bias is a problem for some analyses because the 1B clade is n=1. The IL-8 phenotype and cell shape phenotypes is interesting but complicated**.

**Response:** We agree with the reviewer that the phenotypes measured are complex, but the analyses we performed did allow us to map a portion of the phenotypic variation to specific genetic polymorphisms, which we feel is worth reporting. While there is only a single isolate in clade 1B, the genetic divergence of this isolate from the 1A isolates is similar to that observed between the 2A and 2B isolates (Fig. 4b). Presumably if we had been able to collect and sequence more isolates from the initial timepoint we would have discovered additional 1B isolates, but this does not negate the result that SC4 is quite divergent from the rest of the ancestral isolates.

6) **Method for distinguishing between ancestral and recent diversity (Table 1) is not described and needs at the minimum a phylogenomic analysis if not ancestral reconstruction.**

**Response:** The values in the original Table 1 were the number of unique SNPs and Indels detected within the population compared to the reference (Genbank J99). Table 1 has been edited to remove SNPs/Indels detected in our J99 isolate that differed from the published reference and were shared by all isolates in the collection. Please see our response to reviewer 3, bullet 2.

_____________________________________________________________________

**Response to reviewer 3, bullet 2**: We found 625 SNPs and 130 Indels in our J99 isolate that differ compared to the published reference of J99 (AE001439). A total of 553 of these polymorphic sites were common to all isolates in the collection. We agree with reviewer 3 that including these in Table S1A/Table 1/Text L189 gives an inflated number of ‘total unique SNPs’ and can lead to misinterpretation of the diversity present. This was also expressed by reviewer 2. We have modified Table 1 and Table S1a-b so that variable sites between our J99 isolate and reference that are shared across all isolates were not included in the overall total count. Common SNPs and Indels are now listed in two separate supplementary tables Table S1c and Table S1d. Values have also been changed in the text (L37, L181). Figure 3B and Table S3 have also been altered to reflect the edited SNP total. We have acknowledged the variation in our J99 isolate sequence compared with the published GenBank reference at line 171-174:

“*Our J99 isolate differed from the reference J99 (AE001439) at 755 polymorphic sites (625 SNPs, 130 indels); of these 553 polymorphisms were common to all isolates in the collection (Table S1c-d) and were excluded from downstream analysis.*”

In the discussion at lines 423-429 we explain that these differences may reflect improvements to sequencing methods since the original sequence was published in 1999, differences between our stock of strain J99 compared to that used by Alm et al. in their sequencing project, or mapping errors.

“*We re-sequenced isolate J99, originally isolated from the patient in 1994 and detected 755 polymorphisms differing from the published reference (GenBank, AE001439). The majority (553 polymorphisms) were shared across all isolates, and were therefore excluded from the analysis. These polymorphisms reflect either improvements to sequencing methods since the original sequence was published in 1999, differences in the J99 stock used in our experiment compared to that of Alm et al. (AE001439), or misalignments of short read data to the reference*.”

For the “recent SNPs” part of Table 1, only SNPs absent from ancestral population were counted and reported. Therefore, the diversity reported is not inflated (Total=1,379 SNPs).

______________________________________________________________________

7) **Recent diversity could actually have existed earlier without detection, right? Repeated, different (I think they are unique, but clarify?) mutations in the same gene (Table 2) could result from diversifying selection in that trait but also could result from poor assembly or read-mapping. Recombination in these regions could also confound inference. Please comment on these possibilities.**

**Response**: The values in Table 2 represent unique polymorphisms in the same gene that are present in the sampled recent population and absent in the sampled ancestral population. This is made more clear in the text lines 232-235.

“*Individual genes acquiring the most genetic variation over the six year period were identified. The number of unique nSNPs among the recent isolates detected for each gene were counted and weighted according to the gene length. The genes most highly enriched for unique nSNPs are listed in Table 2 (Table S3).”*

The diversity detected and reported in Table 2 could arise from multiple different mechanisms including de-novo mutation or recombination. We decided to include all polymorphic sites in Table 2 because we predicted polymorphisms introduced by recombination may contribute to some of the observed bacterial phenotypes.

We have now performed additional analysis to identify recombinogenic sites using ClonalFrameML. As we had originally hypothesized, ClonalFrameML predicts that the variation in *cagY* that modified the IL-8 phenotype was introduced by a recombination event. Please see new supplementary Table 9 for a full list of importation events predicted by ClonalFrameML. Please also see our comments to reviewer 3, bullet #1.

______________________________________________________________________

**Response to Reviewer 3, bullet #1**:

In order to identify predicted sites of recombination and assess the impact of recombination on the topology and scale of the Nextstrain tree, we created a recombination-corrected tree using ClonalFrameML(10). This did not change the overall tree topology. However, branch lengths did differ (See new supplementary Fig. S1). ClonalFrameML also calculated the ratio of recombination rate to mutation rate (R/Θ), average import size (δ), and average divergence of imported DNA (ν). We calculated that polymorphisms were roughly 3x more likely to come from recombination than from mutation (r/m=2.95), which was expected since other studies have found recombination to be a major contributor to within host diversity (11). We included this in the text lines 242-250.

“*In order to differentiate polymorphism introduced by recombination from repeated mutation, we bioinformatically identified importation sites with ClonalFrameML [54]. A total of 228 importation events across 52 genes were detected in total (Table S9). All of the genes accumulating an excess of nSNPs listed in Table 2 had at least one detectable recombination event, except jhp_0929, a gene of unknown function located within a large highly variable TnPZ associated genomic region called the plasticity zone [55]. The average import size (δ) was 136bp and divergence of import (ν) was 3.9x10^-2^ subs/site, suggesting imports were from a genetically distinct strain either not sampled or not present at the time of sampling.”*

Typically, estimates of within host recombination/mutation rates (r/m) are highly variable person-to-person and have been hypothesized to depend on the presence of mixed infection (1). This is discussed in the introduction as requested by reviewer 2, bullet 1 (lines 121-131).

“*Whole genome sequencing of sequential isolates from individual infections detected accumulation of point mutations and frequent recombination among co-colonizing isolates. Within host diversity is acquired in a clock-like manner with average estimated mutation rate of 2.5 x 10^-5^ substitutions/site/year. However, in the majority of individuals recombination is the main driver of within-host diversification, which introduces changes at a median rate of 5.5 × 10^−5^ substitutions/initiation site/year [45,46]. The rate of polymorphisms introduced via recombination compared to mutation (r/m rates) vary widely between individuals and likely depends on the presence of multiple co-infecting strains [47,48]. Phylogenetic studies of isolates within a single individual, have observed signatures of diversifying selection in support of selective pressures during chronic stomach colonization [7,37,46].*”

___________________________________________________________________

We agree recent diversity could have existed without detection and have acknowledged this in the manuscript at lines 248-250 and in the discussion lines 449-453:

“*The average import size (δ) was 136bp and divergence of import (ν) was 3.9x10^-2^ subs/site, suggesting imports were from a genetically distinct strain either not sampled or not present at the time of sampling*.”

“*The high number of polymorphic sites within OMPs, including hopQ, sabA, babA, and sabB, were predicted to have accumulated from recombination events and likely originated from a genetically related co-infecting strain, which was either not sampled or not present in the original population.”*

To address whether some of this variation may be due to mis-mapping, we performed sanger sequencing from a few highly variable genes in this table (*sabA*, *hopQ*), and have provided the alignments to the reference in the below figure. This analysis suggest that while there were some mapping errors, diversity detected is not an artifact. Roughly 30 nSNPs were detected between aligned regions of J99 and D1 HopQ, and a total of 11 mutations between aligned regions of J99 and D1 SabA. Sanger sequences showed that the diversity in these genes in some cases may be an under-representation of the diversity present since our analysis only covered regions that aligned well to the reference (see methods lines 541-544).

“*Using J99 ancestral as the reference strain (AE001439), variants were called from raw paired end reads using the Breseq v0.35.0 software with default parameters and SNPs were further validated using default Samtools software suite [69]****.*”**

Some regions of the gene are so divergent that they do not align to J99 (i.e. C-terminus of HopQ, see below). These data support the hypothesis that diversity arose from recombination event(s) due to mixed infection with a highly related strain or gene conversion/duplication event as stated in the text.

**Sanger Sequencing confirms extensive allelic diversity in HopQ and SabA**. (**A**) Complete amino acid sequence alignment of HopQ from J99, D1, and C2. (**B**) Complete amino acid sequence alignment of SabA from J99 and D1. Amino acids highlighted in red are identical, amino acids in blue and black represent polymorphic sites with those in blue representing amino acids that differ but have similar side chains, and those in black representing amino acids with side chains of a different charge.

While variation in OMPs likely has functional consequences, it was not the focus of our current phenotypic analysis and thus we would like to pursue more in-depth analysis of this topic in a future publication.

8) **Fig 2 – how are SVs accounted in your estimate of pairwise nucleotide variation? Looks like difference is driven by enrichment of a more divergent set of isolates from the first sampling point.**

**Response**: While we did detect structural variation among isolates with BreSeq, we did not include these events in our pairwise nucleotide calculations. Variants were called relative to J99 and only SNPs with sufficient coverage in both isolates (≥5 reads) were used in the pairwise analysis.

These methods are described in the text as follows lines 571-581 with newly added information about structural variation

“*The number of nucleotide differences per site (genetic distance) between pairs of isolates was calculated using PopGenome (R, v 4.0.2) using the merged sample variant call file (VCF) file available on Github (nucleotide diversity, π, [71], PopGenome, [72]). All sites that did not align to the reference genome, J99, or had read depth <5 were excluded from the analysis, which removed missing or ambiguous sites resulting from structural variation or mis-mapping. The unique number of shared sites for each pair was calculated using the BEDtools intersect function (reported in Table S5) [73]. The total number of nucleotide differences for each pair was derived from the merged sample VCF file with low quality sites filtered according to the read depth parameters above. All detected indels were excluded from this analysis to avoid inflation of genetic distance due to alignment errors within highly repetitive regions [74].”*

**9) Fig 5: panel C is disingenuous. There’s no relationship between numbers of SNPs and IL8 induction, this is very clearly a false correlation where time and likely a few mutations are the causative variable**.

**Response**: The original Fig. 5c panel shows that as pairwise distance increases so does IL-8 divergence. We agree with the reviewer that a few mutations are likely the causative variable and in Fig 6, we have identified the mutations in *cagY* as some of those mutations contributing to this phenotype. However, in looking at the tree in figure 5a we also see that that the phenotype is more complex. Most isolates in group 2A have reduced IL-8 induction compared to isolates in group 2B despite having the same cagY genotype. We have changed this figure to include both between time point comparisons and within time point observations. The between time point comparisons show that as isolates diverge over time their IL8 phenotypes also diverge. The within time point comparisons, which excludes variables including time and likely many other mutations, shows that there are still additional factors that influence the phenotypes within subgroups that we have not identified. This information was added to a new panel 5D and to the text lines 328-331:

“*There is also a weaker correlation between genetic and phenotypic distances of within timepoint pairs, indicating genetic divergence within the population at a single time point may also contribute to the phenotypic heterogeneity observed (Fig. 5d).*”

Reviewer 3:

1) **L269//506-509: The authors chose to use an “agnostic” (their word) approach, i.e. not to distinguish between mutation and polymorphisms introduced by recombination. In my view, this is a very bad choice, since it makes the results (e.g. the frequency of polymorphisms) impossible to compare with other studies, and likewise very hard to interpret. A quick analysis of the sequences shows ample evidence of recombination in the later set of strains. Methods for accurate phylogenetic inference of recombination in bacterial genomes, such as Gubbins or ClonalFrameML, are now available and widely used. Why choose a dendrogram over a phylogenetic tree that would have provided a more accurate representation of the evolutionary relationships between the isolates? Moreover, a brief inspection the VCF file provided on the Github repository indicates that many SNPs present in the recent group of isolates are tightly clustered together, suggesting that a majority of the diversity could actually come from recombination. In any case, more technical information should be provided concerning the construction of the dendrogram (i.e. is it just hierarchical clustering of pairwise SNP count/genetic distance or was a substitution model used?).**

**Response**: Since the bacterial phenotypes observed in this study may have arisen from intra or inter genomic recombination events as well as mutation, we chose not to remove any sites for initial construction of the tree (Fig 4a). However, we agree with the reviewer that there is evidence that much of the genetic diversity in this isolate collection could have arisen from recombination among highly related strains or also via intragenomic recombination. These possibilities have now been acknowledged in the text lines 449-455:

“*The high number of polymorphic sites within OMPs, including hopQ, sabA, babA, and sabB, were predicted to have accumulated from recombination events and likely originated from a genetically related co-infecting strain, which was either not sampled or not present in the original population. High variation in these genes is unsurprising considering paralogous OMPs have been shown to diversify within-host via frequent intra and intergenomic recombination [22]*.”

In order to identify predicted sites of recombination and assess the impact of recombination on the topology and scale of the Nextstrain tree, we created a recombination-corrected tree using ClonalFrameML(10). This did not change the overall topology drastically. However, branch lengths did differ (See new supplementary Fig. S1). ClonalFrameML also calculated the ratio of recombination rate to mutation rate (R/Θ), average import size (δ), and average divergence of imported DNA (ν). We calculated that polymorphisms were roughly 3x more likely to come from recombination than from mutation (r/m=2.95), which was expected since other studies have found recombination to be a major contributor to within host diversity (11). We included this in the text lines 242-250.

“*In order to differentiate polymorphism introduced by recombination from repeated mutation, we bioinformatically identified importation sites with ClonalFrameML [54]. A total of 228 importation events across 52 genes were detected in total (Table S9). All of the genes accumulating an excess of nSNPs listed in Table 2 had at least one detectable recombination event, except jhp_0929, a gene of unknown function located within a large highly variable TnPZ associated genomic region called the plasticity zone [55]. The average import size (δ) was 136bp and divergence of import (ν) was 3.9x10^-2^ subs/site, suggesting imports were from a genetically distinct strain either not sampled or not present at the time of sampling.”*

Typically, estimates of within host recombination/mutation rates (r/m) are highly variable person-to-person and have been hypothesized to depend on the presence of mixed infection (1). This is discussed in the introduction as requested by reviewer 2, bullet 1 (lines 121-131).

“*Whole genome sequencing of sequential isolates from individual infections detected accumulation of point mutations and frequent recombination among co-colonizing isolates. Within host diversity is acquired in a clock-like manner with average estimated mutation rate of 2.5x10^-5^ substitutions/site/year. However, in the majority of individuals recombination is the main driver of within-host diversification, which introduces changes at a median rate of 5.5 × 10^−5^ substitutions/initiation site/year [45,46]. The rate of polymorphisms introduced via recombination compared to mutation (r/m rates) vary widely between individuals and likely depends on the presence of multiple co-infecting strains [47,48]. Phylogenetic studies of isolates within a single individual, have observed signatures of diversifying selection in support of selective pressures during chronic stomach colonization [7,37,46].*”

Using TimeTree implemented in Nextstrain, we were able to calculate the molecular clock rate for the recombination corrected tree using the root-to-tip distance (see new supplementary Fig. S2a-d) (12). We calculated an average rate of substitution of 2.2 x10^-5^ substitutions/site/year, which is in accordance with the median mutation rate 2.5x10^-5^ substitutions/site/year previously calculated for sequentially isolated strains from the same host (Fig. S2d) (2). Computationally identified sites of recombination and gene annotations are included in a supplementary Table S9 and genes with detected importation events are starred in Table S3. We added this to the manuscript lines 268-278 and lines 430-446:

“*The inferred molecular clock rate calculated from a time-scaled tree using the root-to-tip distance (1.35 x 10 ^-4^ subs/site/year) is similar to the molecular clock rate calculated from pairwise comparisons (Fig. 2a, Fig. S2a-b). Expectedly, this rate is much higher than other within-host estimates that exclude predicted recombination events [45]. To explore the impact of recombination on the tree branch scale and topology, we generated a recombination corrected phylogeny using ClonalFrameML (Fig. S1b). We found the branch lengths of the maximum likelihood tree (Fig. 4a, Fig. S1a) were on average 5 times greater than that of the recombination corrected tree, but general topology remained the same (Fig. S1b). The inferred molecular clock rate from the recombination corrected time-scaled tree was 2.2 x10^-5^ subs/site/year, which is consistent with published estimates (Fig. S2d) [45,48,54].”*

“*Previous studies have found within host evolution of H. pylori is shaped both by de-novo mutation and homologous recombination events, with recombination events generating the majority of the overall diversity [60]. Polymorphism introduced by both mechanisms can impact phenotype, therefore with our analysis we reported and explored sequence level diversity acquired by both mechanisms. Others have estimated the within-host mutation rate to be between 6.5-0.5 x 10^-5^ substitutions per site per year by excluding predicted recombination sites in order to make evolutionary inferences [35,44,47,54]. However, it remains debated whether diversity generated via recombination versus mutation can be accurately identified and filtered to reconstruct evolutionary relationships [56,57,58]. Since clustered nucleotide polymorphisms (CNPs), typically excluded from molecular clock rate calculations, were included in this analysis, our overall within-host molecular clock rate of 1.3 x 10^-4^ subs/site/yr is elevated compared with rates based on substitutions from mutation alone [44]. However, when we excluded polymorphism introduced via importation events using ClonalFrameML, we calculated a molecular clock rate of 2.2 x 10^-5^ subs/site/yr, consistent with the other published estimates [44,47]. This analysis also upholds a model of within host diversity arising predominantly from recombination.”*

”

While we think it is appropriate to include all variable sites in our analysis, we have generated a tree where polymorphisms predicted to arise from recombination events were removed from the analysis. The tree was reconstructed using the IQTree tree implemented in Nextstrain, the branch lengths were scaled similar to the ClonalFrameML tree (see below). The topology of the tree was slightly altered, but the clustering of isolates into the subgroups defined in the manuscript (1A, 1B, 2A, and 2B) remained the same. This supports our conclusions that within each time point the population can be differentiated into two distinct subgroups of divergent isolates.

**Comparison of maximum-likelihood tree, recombination corrected phylogeny, and recombination filtered phylogeny**. X-axis represents divergence from common ancestor (substitutions/site) and scale bar with length indicated in red text is shown. Scale of branches is indicated in red. (**A**) The structure of the maximum likelihood tree constructed in Nextstrain (Fig. 4a, <<https://nextstrain.org/community/salama-lab/Hp-J99>>) and (**B**) the recombination corrected phylogeny created in ClonalFrameML with rescaled branch lengths are shown. Tree construction is described in detail in the methods. (**C**) An additional maximum likelihood Nextstrain tree re-built with IQTree excluding sites that were predicted to be imported via a recombination event is shown.

We have now added additional text in the Materials and Methods to clarify how the original nextstrain tree and the new recombination corrected tree was built lines 586-604

“***Construction of the maximum likelihood and recombination-corrected trees***

*SNPs detected across each isolate reported in the merged sample VCF were imported into the reference J99 (AE001439) to create a consensus genome for each of the 39 isolates. The maximum-likelihood tree (Fig. 4a, Fig. 5a, Fig.7a, Fig. S1a) was built in IQtree implemented in Nextstrain v 1.8.1, using a general time reversible (GTR) substitution model. The ancestral root was inferred using joint maximum likelihood algorithm. All datasets, config files, documentation, and scripts used in this analysis or to generate figures are available publicly on our Github page <https://github.com/salama-lab/Hp-J99>. The interactive tree can be found at the Nextstrain community site <*[*https://nextstrain.org/community/salama-lab/Hp-J99*](https://nextstrain.org/community/salama-lab/Hp-J99)*>. To create the recombination corrected tree with rescaled branch lengths, the reference-aligned consensus genomes were aligned using progressiveMauve assuming collinear genomes and a new rescaled tree (Fig. S1b) was generated using ClonalFrameML using all sites in the reference (AE001439) [54,75]. Time scaled trees and molecular clock rates (Fig. S2) were generated with TreeTime implemented within Nextstrain, which approximates divergence times and molecular clock rates using maximum likelihood methods [76]. Figure S1 and branch lengths of the maximum likelihood and recombination corrected trees were compared using the ape and adephylo packages in R, v 4.0.2.”*

**2**) **L189/Table 1/Table S1A. It appears that strain J99 was re-sequenced along with the other isolates. Surprisingly, the new J99 sequence displays hundreds of differences compared to the Genbank sequence of J99. These differences must either be sequencing errors in the reference sequence or in the Illumina data, or the sequences do not come from the same strain. Furthermore, and of even more concern, most the SNPs observed between the “old” and “new” versions of J99 also appear in all the isolates in this study. This raises substantial doubts about all quantitative statements regarding sequence changes during the six years of infection. There are several possible explanations for this, one possibility is that the clone sequenced by Richard Alm and colleagues was substantially different from the current J99. Since the patient apparently did have a mixed infection (since there is so much recombination), this is possible. Alternatively, the old sequence may contain many errors, but then the point of reference for all isolates should be the “new” J99 sequence, not the old one. This issue appears to inflate the number of unique SNPs observed by around 20%, which is very significant. It would probably be best to complete the new genome sequence of J99 (e.g. by adding PacBio or Oxford Nanopore long reads) and to use this as a reference for the analysis. This is a major issue affecting the validity of all comparisons and must be addressed.**

**Response**: We found 625 SNPs and 130 Indels in our J99 isolate that differ compared to the published reference of J99 (AE001439). A total of 553 of these polymorphic sites were common to all isolates in the collection. We agree with reviewer 3 that including these in Table S1A/Table 1/Text L189 gives an inflated number of ‘total unique SNPs’ and can lead to misinterpretation of the diversity present. This was also expressed by reviewer 2. We have modified Table 1 and Table S1a-b so that variable sites between our J99 isolate and reference that are shared across all isolates were not included in the overall total count. Common SNPs and Indels are now listed in two separate supplementary tables Table S1c and Table S1d. Values have also been changed in the text (L37, L181). Figure 3B and Table S3 have also been altered to reflect the edited SNP total. We have acknowledged the variation in our J99 isolate sequence compared with the published GenBank reference at line 171-174:

“*Our J99 isolate differed from the reference J99 (AE001439) at 755 polymorphic sites (625 SNPs, 130 indels); of these 553 polymorphisms were common to all isolates in the collection (Table S1c-d) and were excluded from downstream analysis*.”

In the discussion at lines 423-429 we explain that these differences may reflect improvements to sequencing methods since the original sequence was published in 1999, differences between our stock of strain J99 compared to that used by Alm et al. in their sequencing project, or mapping errors.

“*We re-sequenced isolate J99, originally isolated from the patient in 1994 and detected 755 polymorphisms differing from the published reference (GenBank, AE001439). The majority (553 polymorphisms) were shared across all isolates, and were therefore excluded from the analysis. These polymorphisms reflect either improvements to sequencing methods since the original sequence was published in 1999, differences in the J99 stock used in our experiment compared to that of Alm et al. (AE001439), or misalignments of short read data to the reference*.*”*

For the “recent SNPs” part of Table 1, only SNPs absent from ancestral population were counted and reported. Therefore, the diversity reported is not inflated (Total=1,379 SNPs).

3) **L191: Both mutation and recombination are considered here, thus using “polymorphism” instead of “mutation” would be more accurate.**

**Response**: We have made corrections to this line and other instances throughout the manuscript where mutation was used to describe SNPs.

***4*) L227-228: How different is the pool of within-host variable OMPs between the old and recent group of isolates?**

**Response***:* There were some OMPs that exhibited diversity at both time points but diverged during the 6 years and there were some that were identical to the reference at the first time point and then acquired diversity throughout infection. We have added two new columns to Table 2 to show the estimated number of alleles in the population for both the ancestral and recent populations.

***5****)* **L258-259: Are these four isolates all from the recent group? The 23S rRNA is typically fairly conserved. How do you explain that polymorphisms related to antibiotic resistance appeared at a significant frequency in the population with no known history of antibiotic treatment?**

**Response**: Antibiotic testing of this collection of isolates and the 23S mutation is primarily discussed in the original analysis of these isolates by Israel et al in 2001 (13). All of these isolates were from the recent group (3 antral, one duodenal). It is possible those mutations were present in the original population and just weren’t sampled or that the patient did have an exposure to clarithromycin that was not documented, and did not eradicate infection. Also see our comments to reviewer 2 about sampling bias in major revision bullet 1.

**6**) **Figure 4/5/6/7: The unit used on the x-axis may not be correct. On the NextStrain platform, the scale indicates “Divergence”, which seems to correspond to the accumulation of changes since the common ancestor and not a rate like “substitution per site per year” seems to indicate.**

**Response**: We agree with the reviewer that the x-axis should be changed to coincide with the figure displayed on the nextstrain platform. Nextstrain does infer an ancestral root, therefore the x-axis label should be divergence (substitutions/site) from the common ancestor. We corrected this on all figures and figure legends. We also added a description of the tree construction as reported in bullet 1 to the methods at lines 586-604:

**“*Construction of the maximum likelihood and recombination-corrected trees***

*SNPs detected across each isolate reported in the merged sample VCF were imported into the reference J99 (AE001439) to create a consensus genome for each of the 39 isolates. The maximum-likelihood tree (Fig. 4a, Fig. 5a, Fig.7a, Fig. S1a) was built in IQtree implemented in Nextstrain v 1.8.1, using a general time reversible (GTR) substitution model. The ancestral root was inferred using joint maximum likelihood algorithm. All datasets, config files, documentation, and scripts used in this analysis or to generate figures are available publicly on our Github page <https://github.com/salama-lab/Hp-J99>. The interactive tree can be found at the Nextstrain community site <*[*https://nextstrain.org/community/salama-lab/Hp-J99*](https://nextstrain.org/community/salama-lab/Hp-J99)*>. To create the recombination corrected tree with rescaled branch lengths, the reference-aligned consensus genomes were aligned using progressiveMauve assuming collinear genomes and a new rescaled tree (Fig. S1b) was generated using ClonalFrameML using all sites in the reference (AE001439) [54,75]. Time scaled trees and molecular clock rates (Fig. S2) were generated with TreeTime implemented within Nextstrain, which approximates divergence times and molecular clock rates using maximum likelihood methods [76]. Figure S1 and branch lengths of the maximum likelihood and recombination corrected trees were compared using the ape and adephylo packages in R, v 4.0.2.*”

***7****)* **Figure 4/5/6/7: Isolates from the duodenum, corpus and antrum regions appear in both subgroups 2A and 2B. This should be discussed***.*

The lack of “region specific adaptation” is discussed at L304-L309:

“*Consistent with this finding, both recent subgroups (yr 2000) contain isolates from all three biopsy locations and nearest neighbors on the tree often come from different biopsies (Fig. 4a). The formation of distinct subgroups within a population of isolates collected from a single time point, suggests the possibility of niche level adaptation, but these distinct niches must be present in all regions of the stomach sampled.*”

and is addressed in the discussion L466: “*our data do not support subgroup divergence by anatomic region in this individual.*”

8) **Figure 4/5/6/7: The common ancestor of the recent group of isolates appears to only date back to a few months before the sampling date (using the year scale on the Nextstrain platform). How is it explained if the population has been evolving from the population of 6 years ago without a major bottleneck?**

**Response:** Without creating a recombination corrected tree, variation introduced into the population through recombination would be a major confounder in calculation of the time to most recent common ancestor (see new supplemental figure Fig. S2a). Typically, low TMRCA are observed in phylogenetic studies of within host evolution of *H pylori*. This finding is likely due to strong selection or bottlenecks from antibiotic treatment or immune pressure (1,4). We are not claiming absence of major bottlenecks, but just that we can infer antibiotics were likely not the main driver of a bottleneck due to what is known about this patient’s history. We addressed some of these concerns with the updated time tree in the new supplemental Fig. S2c. From this figure we can calculate the rough approximation of the TMRCA from the scaled (recombination corrected) time tree, which is approximately 1 year for the ancestral group and 1.5 years for the recent group. This has been added to the manuscript lines 278-282:

“*We estimated a time to most recent common ancestor (TMRCA) of 1 year for the ancestral population and 1.5 years for the recent population. Low TMRCA are typically observed in phylogenetic studies of within host evolution of H. pylori; and in the absence of antibiotic treatment, this is thought to occur as a result of immune pressure [37,47]*.*”*

Minor Revisions from Reviewer 1, 2, and 3.

Reviewer 1:

1) **Table 2: How many SNPs were seen per individual Hp gene?**

**Response:** Please see new Table 2. We added a new column to show the number of predicted alleles within the population.

2) **Just one SNP per Hp strain and gene or multiple SNPs in any particular gene, e.g. hopQ?**

**Response:** We have clarified in the manuscript at line 232-235 that the table represents unique mutations across all isolates. We have also amended Table 2 to include a column indicating the number of unique alleles in the population estimated from the sequencing data.

“*Individual genes acquiring the most genetic variation over the six year period were identified. The number of unique nSNPs among the recent isolates detected for each gene were counted and weighted according to the gene length. The genes most highly enriched for unique nSNPs are listed in Table 2 (Table S3).*”

**3**) **If multiple SNPs, could they be the result of intrachromosomal recombination?**

**Response***:* This was addressed in comments to reviewer 2 under major revisions bullet 7.

_____________________________________________________________________

**Response to reviewer 2, bullet 7**: The values in Table 2 represent unique polymorphisms in the same gene that are present in the sampled recent population and absent in the sampled ancestral population. This is made more clear in the text lines 232-235.

“*Individual genes acquiring the most genetic variation over the six year period were identified. The number of unique nSNPs among the recent isolates detected for each gene were counted and weighted according to the gene length. The genes most highly enriched for unique nSNPs are listed in Table 2 (Table S3).”*

The diversity detected and reported in Table 2 could arise from multiple different mechanisms including de-novo mutation or recombination. We decided to include all polymorphic sites in Table 2 because we predicted polymorphisms introduced by recombination may contribute to some of the observed bacterial phenotypes.

We have now performed additional analysis to identify recombinogenic sites using ClonalFrameML. As we had originally hypothesized, ClonalFrameML predicts that the variation in *cagY* that modified the IL-8 phenotype was introduced by a recombination event. Please see new supplementary Table 9 for a full list of importation events predicted by ClonalFrameML. Please also see our comments to reviewer 3, bullet #2.

We agree recent diversity could have existed without detection and have acknowledged this in the manuscript at lines 248-250 and in the discussion lines 449-453:

“*The average import size (δ) was 136bp and divergence of import (ν) was 3.9x10^-2^ subs/site, suggesting imports were from a genetically distinct strain either not sampled or not present at the time of sampling*.”

“*The high number of polymorphic sites within OMPs, including hopQ, sabA, babA, and sabB, were predicted to have accumulated from recombination events and likely originated from a genetically related co-infecting strain, which was either not sampled or not present in the original population.”*

To address whether some of this variation may be due to mis-mapping, we performed Sanger sequencing from a few highly variable genes in this table (*sabA*, *hopQ*), and have provided the alignments to the reference in the figure below. This analysis suggest that while there were some mapping errors, diversity detected is not an artifact. Roughly 30 nSNPs were detected between aligned regions of J99 and D1 HopQ, and a total of 11 mutations between aligned regions of J99 and D1 SabA (see below figure). Sanger sequences showed that the diversity in these genes in some cases may be an under-representation of the diversity present since our analysis only covered regions that aligned well to the reference (see methods lines 541-544).

“*Using J99 ancestral as the reference strain (AE001439), variants were called from raw paired end reads using the Breseq v0.35.0 software with default parameters and SNPs were further validated using default Samtools software suite [69]****.*”**

Some regions of the gene are so divergent that they do not align to J99 (i.e. C-terminus of HopQ, see below). These data support the hypothesis that diversity arose from recombination event(s) due to mixed infection with a highly related strain or gene conversion/duplication event as stated in the text.

______________________________________________________________________

**4**) **In Fig. 5 you mention a > 20% increase, but in Fig. 9 you refer to >20% decrease. Please clarify***.*

**Response**: This is a typo that has been corrected to read >20% increase in both figures.

5) **How do you compare the mutation rates to that in previously published papers from isolates of chronically infected patients?**

**Response**: We calculated the mutation rates as requested by reviewers 2 and 3 and have made comparisons to other studies in the discussion. Please see supplementary figure S2 and lines 268-282 and 430-446 in the manuscript.”

“*The inferred molecular clock rate calculated from a time-scaled tree using the root-to-tip distance (1.35 x 10 ^-4^ subs/site/year) is similar to the molecular clock rate calculated from pairwise comparisons (Fig. 2a, Fig. S2a-b). Expectedly, this rate is much higher than other within-host estimates that exclude predicted recombination events [45]. To explore the impact of recombination on the tree branch scale and topology, we generated a recombination corrected phylogeny using ClonalFrameML (Fig. S1b). We found the branch lengths of the maximum likelihood tree (Fig. 4a, Fig. S1a) were on average 5 times greater than that of the recombination corrected tree, but general topology remained the same (Fig. S1b). The inferred molecular clock rate from the recombination corrected time-scaled tree was 2.2 x10^-5^ subs/site/year, which is consistent with published estimates (Fig. S2d) [45,48,54]. We estimated a time to most recent common ancestor (TMRCA) of 1 year for the ancestral population and 1.5 years for the recent population. Low TMRCA are typically observed in phylogenetic studies of within host evolution of H pylori; and in the absence of antibiotic treatment, this is thought to occur as a result of immune pressure [37,48].”*

*“Previous studies have found within host evolution of H. pylori is shaped both by de-novo mutation and homologous recombination events, with recombination events generating the majority of the overall diversity [60]. Polymorphism introduced by both mechanisms can impact phenotype, therefore with our analysis we reported and explored sequence level diversity acquired by both mechanisms. Others have estimated the within-host mutation rate to be between 6.5-0.5x10^-5^ substitutions per site per year by excluding predicted recombination sites in order to make evolutionary inferences [35,45,48,54]. However, it remains debated whether diversity generated via recombination versus mutation can be accurately identified and filtered to reconstruct evolutionary relationships [56,57,58]. Since clustered nucleotide polymorphisms (CNPs), typically excluded from molecular clock rate calculations, were included in this analysis, our overall within-host molecular clock rate of 1.3x10^-4^ subs/site/yr is elevated compared with rates based on substitutions from mutation alone [45]. However, when we excluded polymorphism introduced via importation events using ClonalFrameML, we calculated a molecular clock rate of 2.2x10^-5^ subs/site/yr, consistent with the other published estimates [45,48]. This analysis also upholds a model of within host diversity arising predominantly from recombination.”*

Reviewer 2:

1) **66 “sub niches” awkward and not a standard term.**

**Response**: We have changed all instances of this in the text to “niches.”

2) **77 please provide specific details about what’s known about the extent of diversification of H pylori during a typical infection. Important for setting baseline**

**Response**: This addressed in the major revision reviewer #2 bullet 1. More background was added to the introduction lines 121-131.

“*Whole genome sequencing of sequential isolates from individual infections detected accumulation of point mutations and frequent recombination among co-colonizing isolates. Within host diversity is acquired in a clock-like manner with average estimated mutation rate of 2.5x10^-5^ substitutions/site/year. However, in the majority of individuals recombination is the main driver of within-host diversification, which introduces changes at a median rate of 5.5 × 10^−5^ substitutions/initiation site/year [45,46]. The rate of polymorphisms introduced via recombination compared to mutation (r/m rates) vary widely between individuals and likely depends on the presence of multiple co-infecting strains [47,48]. Phylogenetic studies of isolates within a single individual, have observed signatures of diversifying selection in support of selective pressures during chronic stomach colonization [7,37,46].”*

3) **81-83 same comment about cagY allelic variation – how much?**

**Response:** The amount of variation differs between individuals but variation in cagY has been shown to be increased by expansion and contraction of the repeat region. CagY variation is discussed at lines 80-82 and in the discussion lines 475-478.

“*Cag PAI encoded genes, cagA and cagY exhibit significant allelic variation between individuals and have been identified as targets of positive selection within the global population [12]*.”

“*Recombination within the middle repeat region of cagY, resulting in expansion or contraction of repeats, occurs frequently in short-term animal infections and in humans [15]. We observed modulation of IL-8 induction mediated by T4SS function through mutation and/or recombination without expansion or contraction of cagY repeats.*”

4) **129 what’s the quality of J1 genome? How was it produced? You’ve now resequenced it effectively – how many calls are shared, derived and thus actually in the J1 isolate?**

**Response:** J99 resequencing data has been addressed in the major revisions of Table 1 and in lines 171-174. See comments to reviewer 3, bullet 2.

______________________________________________________________________

**Response to reviewer 3, bullet 2:**

We found 625 SNPs and 130 Indels in our J99 isolate that differ compared to the published reference of J99 (AE001439). A total of 553 of these polymorphic sites were common to all isolates in the collection. We agree with reviewer 3 that including these in Table S1A/Table 1/Text L189 gives an inflated number of ‘total unique SNPs’ and can lead to misinterpretation of the diversity present. This was also expressed by reviewer 2. We have modified Table 1 and Table S1a-b so that variable sites between our J99 isolate and reference that are shared across all isolates were not included in the overall total count. Common SNPs and Indels are now listed in two separate supplementary tables Table S1c and Table S1d. Values have also been changed in the text (L37, L181). Figure 3B and Table S3 have also been altered to reflect the edited SNP total. We have acknowledged the variation in our J99 isolate sequence compared with the published GenBank reference at line 171-174:

“*Our J99 isolate differed from the reference J99 (AE001439) at 755 polymorphic sites (625 SNPs, 130 indels); of these 553 polymorphisms were common to all isolates in the collection (Table S1c-d) and were excluded from downstream analysis.”*

In the discussion at lines 423-429 we explain that these differences may reflect improvements to sequencing methods since the original sequence was published in 1999, differences between our stock of strain J99 compared to that used by Alm et al. in their sequencing project, or mapping errors.

“*We re-sequenced isolate J99, originally isolated from the patient in 1994 and detected 755 polymorphisms differing from the published reference (GenBank, AE001439). The majority (553 polymorphisms) were shared across all isolates, and were therefore excluded from the analysis. These polymorphisms reflect either improvements to sequencing methods since the original sequence was published in 1999, differences in the J99 stock used in our experiment compared to that of Alm et al. (AE001439), or misalignments of short read data to the reference.”*

For the “recent SNPs” part of Table 1, only SNPs absent from ancestral population were counted and reported. Therefore, the diversity reported is not inflated (Total=1,379 SNPs).

______________________________________________________________________

5) **176 92% ANI is beyond the level of species distinction, even genus – this seems inconceivable. Sequencing and mapping details need to be provided up front to justify this claim**

**Response**: We were referencing the analysis published by Alm et al. comparing the two strains (14). Alm et al., calculated 92% ANI between orthologous genes. For context, this is comparing all genes not just the core genome. Our analysis also considered all genes. Orthologous outer-membrane proteins, for example, have a high degree of variability between genetically distinct strains.

6) **184-188 writing is awkward**

**Response**: We have reworded at line 185-187: “*Depletion of indels within coding regions likely reflects purifying selection due to high potential of indels to disrupt gene function by introducing frameshifts.*”

7) **223: “functional classes assigned by the microbial genome database (MGDB) add reference.” What do you mean? Definitely add the reference!**

We added the MGDB reference.

8) **230 you state others have found variation in OMPs but don’t provide references. Be more specific about the kind and consequences of this variation***.*

**Response**: The following text and references were added at line 216-218.

“*This was expected since other studies that have looked at within host populations have shown OMPs accumulate diversity due to frequent inter and intragenomic recombination [7,22,45].”*

9) **Fig 3 statistical enrichment of functional categories should be conservative because of numerous potential biases; omit those at p=0.03.**

**Response:** We have eliminated MGDB categories reported in the figure above a p-value of 0.03. Please see updated Figure 3.

10) **263 “but no mutations indicative of additional antibiotic resistance were discovered.” Vague. Describe methods esp in light of the broad challenge to identify AMR determinants from genomes.**

**Response:** A comprehensive list of genes involved in antibiotic resistance was downloaded from PATRIC. This list is compiled from multiple different databases and indicates PubMed ID references. We have added the following to text L228-231 and the methods L566-568.

“*To validate, we queried our sequence data for polymorphisms known to confer antibiotic resistance listed in the Pathosystems Resource Integration Center (PATRIC) database. Using these methods, no additional polymorphisms indicative of antibiotic resistance were discovered.”*

“*To identify markers of antimicrobial resistance, polymorphisms detected in the collection were screen against a list of antimicrobial resistance genes downloaded from PATRIC <https://www.patricbrc.org >.”*

Reviewer 3**:**

1) **L676: It is not quite clear how experimental variation was assessed. Were multiple tests performed for every individual isolate? What are “pooled replicates from two independent experiments”?**

**Response**: We will change the language in the methods at line 641 to “*at least two biological replicates where each isolate was assayed with triplicate wells*”

***2*) Fig. 1: The information contained in Fig. 1 is also contained in the text, consider deleting it to conserve space.**

**Response:** Fig 1 might not be completely necessary, but we are choosing to leave it in because it provides a visual reference of the basic stomach anatomy and sampling scheme over the 6 year period.

***3****)* **The reference section needs careful checking for completeness and formatting of citations.**

**Response**: We have revised the citations.

**New Figures:**

**Updated Table 1:**

| **Table 1. Summary of unique SNPs, Indels detected by WGS among all the isolates (n=39) and in the subset of recent isolates (n=27, yr 2000)** | | | | | | | | | | |
| --- | --- | --- | --- | --- | --- | --- | --- | --- | --- | --- |
|  |  | **Total^a^** |  | **Coding^a^** | | **nS^b^** | | **S^b^** |  | **Intergenic^a^** |
| **Total SNPs** |  | 1,767 |  | 1,632 |  | | 704 | 928 |  | 135 |
|  |  |  |  |  |  | |  |  |  |  |
| **Recent SNPs** |  | 1,379 |  | 1,270 |  | | 536 | 734 |  | 109 |
|  |  |  |  |  |  | |  |  |  |  |
|  |  | **Total^a^** |  | **Coding^a^** | |  | |  |  | **Intergenic^a^** |
| **Total Indels** |  | 485 |  | 298 | |  | |  |  | 187 |
|  |  |  |  |  |  | |  |  |  |  |
| **Recent Indels** |  | 372 |  | 231 | |  | |  |  | 141 |
|  |  |  |  |  |  | |  |  |  |  |

**^a^**Unique events are labeled as either coding or intergenic. **^b^**Events within coding regions are further subdivided into nonsynonymous (nS) or synonymous (S) categories.

**Updated Table 2:**

| **Gene ID^a^** | **Annotation^a^** | **MGDB function^a^** | **Unique nSNPs** | **Z-scores^b^** | **Estimated no. of alleles^c^** | |
| --- | --- | --- | --- | --- | --- | --- |
|  |  |  |  |  | **Ancestral** | **Recent** |
| jhp1300 |  | Unknown | 18 | 21.52 | 2 | 19 |
| jhp1103 | *hopQ* | Outer membrane protein | 45 | 14.89 | 1 | 11 |
| jhp1068 | *birA* | Biotin protein ligase | 11 | 10.96 | 1 | 1 |
| jhp0659 | *sabB* | Outer membrane protein | 27 | 8.94 | 1 | 13 |
| jhp0303 |  | Translocase subunit | 3 | 7.9 | 1 | 1 |
| jhp1096 | *glnP_1* | Transport and binding proteins | 8 | 7.73 | 1 | 1 |
| jhp1409 |  | Unknown | 44 | 7.39 | 2 | 19 |
| jhp0302 | *argS* | Arginine-tRNA ligase | 18 | 6.98 | 1 | 1 |
| jhp0336 |  | Unknown | 19 | 5.07 | 1 | 1 |
| jhp1097 | *glnP_2* | ABC transporter permease | 5 | 4.63 | 1 | 1 |
| jhp0634 |  | Unknown | 7 | 4.29 | 1 | 1 |
| jhp1102 |  | Guanine permease | 9 | 4.26 | 1 | 1 |
| jhp0833 | *babA* | Outer membrane protein | 15 | 4.16 | 2 | 4 |
| jhp0662 | *sabA* | Outer membrane protein | 13 | 4.12 | 4 | 5 |
| jhp0929 |  | Unknown | 3 | 4.1 | 1 | 3 |

**^a^**The top 15 annotated genes with Z-scores > 4 for number of nSNPs uniquely acquired in the yr 2000 group of isolates within single genes are shown. **^b^**Z-scores were calculated using number of counts per gene normalized according to gene length. ^c^The estimated number of alleles (see Materials and Methods) among 27 isolates in the recent population (2000) and 12 isolates in the ancestral population (1994) are shown. The methods used to estimate the number of alleles are reported in the methods section.

**Updated Figure 5:**

**Updated Fig S1**

**Figure S1. Comparison of maximum-likelihood tree and recombination corrected phylogeny have altered branch scale but the same overall topology**. X-axis represents divergence from common ancestor (substitutions/site). Length scale of branches is indicated in red. (**A**) The maximum likelihood tree constructed in Nextstrain (Fig. 4a, <<https://nextstrain.org/community/salama-lab/Hp-J99>>) and (**B**) the recombination corrected phylogeny created in ClonalFrameML with rescaled branch lengths are shown. Tree construction is described in detail in the Materials and Methods.

**Figure S2. Molecular clock rates based on divergence from the ancestral root differ between time-scaled maximum likelihood tree and recombination corrected phylogeny**. X-axis indicates the date in years and Y-axis **(B, D**) indicates divergence (substitutions/site) from the ancestral root. (**A**) A time-scaled version of the maximum likelihood tree with (**B**) molecular clock rates inferred from the root-to-tip distance using TreeTime [76]. (**C-D**) Shows time-scaled tree (**C**) and inferred molecular clock for the recombination corrected tree (**D**).

**References:**

1.	Didelot X, Nell S, Yang I, Woltemate S, Van Der Merwe S, Suerbaum S. Genomic evolution and transmission of Helicobacter pylori in two South African families. Proc Natl Acad Sci U S A. 2013;110(34):13880–5.

2.	Kennemann L, Didelot X, Aebischer T, Kuhn S, Drescher B, Droege M, et al. Helicobacter pylori genome evolution during human infection. Proc Natl Acad Sci [Internet]. 2011;108(12):5033–8. Available from: http://www.pnas.org/cgi/doi/10.1073/pnas.1018444108

3.	Linz B, Windsor HM, McGraw JJ, Hansen LM, Gajewski JP, Tomsho LP, et al. A mutation burst during the acute phase of Helicobacter pylori infection in humans and rhesus macaques. Nat Commun [Internet]. 2014;5(May):1–8. Available from: http://www.nature.com/doifinder/10.1038/ncomms5165

4.	Ailloud F, Didelot X, Woltemate S, Pfaffinger G, Overmann J, Bader RC, et al. Within-host evolution of Helicobacter pylori shaped by niche-specific adaptation, intragastric migrations and selective sweeps. Nat Commun. 2019;10(1).

5.	Falush D, Kraft C, Taylor NS, Correa P, Fox JG, Achtman M, et al. Recombination and mutation during long-term gastric colonization by Helicobacter pylori: Estimates of clock rates, recombination size, and minimal age. Proc Natl Acad Sci [Internet]. 2001;98(26):15056–61. Available from: http://www.pnas.org/cgi/doi/10.1073/pnas.251396098

6.	Solnick J V., Hansen LM, Salama NR, Boonjakuakul JK, Syvanen M. Modification of Helicobacter pylori outer membrane protein expression during experimental infection of rhesus macaques. Proc Natl Acad Sci U S A. 2004;101(7):2106–11.

7.	Nei M, Li WH. Mathematical model for studying genetic variation in terms of restriction endonucleases. Proc Natl Acad Sci U S A. 1979;76(10):5269–73.

8.	Pfeifer B, Wittelsbürger U, Ramos-Onsins SE, Lercher MJ. PopGenome: An efficient swiss army knife for population genomic analyses in R. Mol Biol Evol. 2014;31(7):1929–36.

9.	Quinlan AR, Hall IM. BEDTools: A flexible suite of utilities for comparing genomic features. Bioinformatics. 2010;26(6):841–2.

10.	Didelot X, Wilson DJ. ClonalFrameML: Efficient Inference of Recombination in Whole Bacterial Genomes. PLoS Comput Biol. 2015;11(2):1–18.

11.	Dorer MS, Sessler TH, Salama NR. Recombination and DNA Repair in Helicobacter pylori. Annu Rev Microbiol. 2008;23(1):1–7.

12.	Sagulenko P, Puller V, Neher RA. TreeTime: Maximum-likelihood phylodynamic analysis. Virus Evol. 2018;4(1):1–9.

13.	Israel DA, Salama N, Krishna U, Rieger UM, Atherton JC, Falkow S, et al. Helicobacter pylori genetic diversity within the gastric niche of a single human host. Proc Natl Acad Sci U S A [Internet]. 2001;98(25):14625–30. Available from: http://www.pnas.org/content/98/25/14625.abstract%5Cnhttp://www.pnas.org/content/98/25/14625.short

14.	Alm RA, Trust TJ. Analysis of the genetic diversity of Helicobacter pylori: The tale of two genomes. J Mol Med. 1999;77(12):834–46.

15.	Talarico S. Regulation of Helicobacter pylori adherence by gene conversion. Mol Microbiol. 2012;84(6):1050–61.
